# Supplementary material for: Effects of 10-Day Complete Fasting on Physiological Homeostasis, Nutrition and Health Markers in Male Adults
Source: Nutrients. 2022 Sep 18;14(18):3860. doi: 10.3390/nu14183860 (PMC9503095; doi:10.3390/nu14183860)
Supplement: Supplementary file 1 [file nutrients-14-03860-s001.zip › nutrients-1876445-supplementary.pdf]

Supplement table and figure.

Table S1 the results of 17 blood routine parameters

	BF	CF3	CF6	CF9	CR3	FR5
WBC	6.01±1.71	6.61±2.31	6.01±1.81	6.01±2.11	5.01±1.21	5.31±1.31
LYM	2.01±0.51	2.01±0.71	1.91±0.61	1.91±0.51	1.91±0.41	1.81±0.51
LYM%	34.01±5.71	30.41±6.71	31.51±5.81	34.11±8.61	38.31±8.71	35.81±6.81
EO	0.21±0.21	0.11±0.11	0.21±0.21	0.21±0.21	0.21±0.21	0.21±0.11
EO%	3.61±2.41	2.41±1.91	3.11±3.11	3.61±3.11	4.31±3.01	4.21±2.51
BASO	0.01±0.01	0.01±0.01	0.01±0.01	0.11±0.01	0.01±0.01	0.01±0.01
BASO%	0.61±0.21	0.51±0.21	0.71±0.31	0.91±0.31	0.71±0.31	0.41±0.51
MCV	91.21±7.61	91.31±8.21	90.81±8.11	88.31±6.81	87.61±7.41	92.21±6.91
MCH	31.21±2.81	31.31±2.71	30.51±3.81	29.91±2.41	30.41±2.41	30.01±2.31
MCHC	342.21±10.11	342.71±13.31	345.51±13.01	338.81±11.41	346.81±12.21	325.51±9.11
RDW-SD	41.61±6.81	41.41±7.01	41.31±6.21	39.21±4.91	39.21±5.31	42.81±5.31
RDW-CV	12.51±0.91	12.31±0.91	12.41±0.71	12.11±0.61	12.31±0.71	13.01±0.91
PLT	247.21±55.71	261.51±40.91	277.51±41.41	280.51±45.01	235.51±47.01	240.31±54.71
PDW	12.61±1.21	12.61±1.31	13.01±1.21	12.81±1.31	10.31±1.11	13.81±1.41
MPV	10.61±0.61	10.61±0.61	10.81±0.51	10.61±0.51	9.51±0.61	11.11±0.61
PLCR	29.81±4.91	29.71±4.81	31.61±4.61	30.21±4.31	21.81±4.71	33.31±4.11
PCT	0.31±0.11	0.31±0.01	0.31±0.01	0.31±0.01	0.21±0.01	0.31±0.11

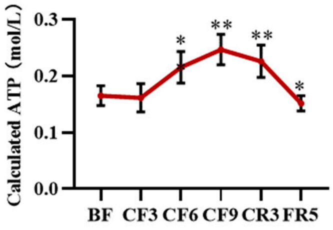

Figure S1 the calculated ATP level during the experiment. * $p<0.05$, ** $p<0.01$ VS. BF; n=13. BF: before fasting, CF: complete fasting, CR: calorie restriction, FR: fully recovery, the number presents days.
